# Intrinsic and Extrinsic Quality Attributes of Fresh and Semi-Hard Goat Cheese from Low- and High-Input Farming Systems

**DOI:** 10.3390/ani10091567

**Published:** 2020-09-03

**Authors:** Annalaura Lopez, Mauro Vasconi, Monica Battini, Silvana Mattiello, Vittorio Maria Moretti, Federica Bellagamba

**Affiliations:** 1 Department of Veterinary Medicine, Università degli Studi di Milano, Via dell’Università, 6, 26900 Lodi, Italy; annalaura.lopez@unimi.it (A.L.); vittorio.moretti@unimi.it (V.M.M.); federica.bellagamba@unimi.it (F.B.); 2 Department of Agricultural and Environmental Sciences, Production, Landscape, Agroenergy, Università degli Studi di Milano, Via Celoria, 2, 20133 Milano, Italy; monica.battini@unimi.it (M.B.); silvana.mattiello@unimi.it (S.M.)

**Keywords:** animal welfare, fatty acids, goat cheese, livestock production system

## Abstract

**Simple Summary:**

Modern consumers are continuously more interested in animal food product qualities that could be considered both extrinsic and intrinsic. Goat products suitably match with these interests because of their functional properties, the ethics of the related livestock production, the environmental impact of production, and animal welfare issues. Through a combination of laboratory analysis and in-field observation, we found that the nutritional quality of goat cheese is affected by the livestock production system, but this is not always true when considering the intrinsic quality related to animal welfare aspects. For this reason, we suggest that more detailed information on different quality traits should be provided to consumers in order to allow them to reach a complete view of product quality and, consequently, to achieve more conscious food consumption.

**Abstract:**

In this study, we investigated the lipid composition of fresh and semi-hard goat cheese produced in three Italian farms as well as the welfare assessment of goats reared in these farms. The fatty acid (FA) profile of cheese samples were found to be strictly related to the livestock system. Cheese collected from farms in which goats were allowed to graze and were fed diets with a higher forage/concentrate (F/C) ratio showed a FA profile represented by higher contents of health-promoting fatty acids. In the same samples, the health lipid indices showed the most favorable values. Conversely, cheese samples collected from a conventional-lowland farm, where goats were fed with higher amounts of concentrates and lower F/C ratio, presented a lower nutritional quality, characterized by the worst results for what concerns the health lipid indices. Then, we built a multivariate model able to discriminate samples coming from farms managed by a low-input system from those coming from farm managed by a high-input system. The comparison of animal welfare measurements and fatty acids data showed that a better intrinsic quality of low-input farms did not always correspond to better extrinsic quality, suggesting that the information on the livestock system is not always enough to provide consumers with complete awareness of the total product quality.

## 1. Introduction

Nowadays, a significant interest in dairy goat products has been growing among both the scientific community and consumers, due to spreading knowledge about their functional properties and high nutritional value [1,2,3]. Moreover, dairy goat products finely match with the interests of present-day consumers, especially regarding the ethics of livestock production, environmental impact, and animal welfare issues. These aspects are included in a broader food quality concept that considers not only the product’s intrinsic quality attributes, but also its extrinsic attributes. As stated by Massaglia et al. [4] during recent years, goat products have gradually been associated with consumer perception of innate quality because of their status of being sustainable, traditional, and health products. These features have a fundamental importance for the assessment of these products to the “functional food” business, in which Italy is estimated to generate 11% of the total revenues of the total European market share [5]. Nevertheless, Italian consumers are still confused about what functional food products are, even if they are strongly conscious of the existing link between the diet and their health and they have demonstrated increasing interest toward the health implications of their food choices [6].

According to FAOSTAT (Food and Agriculture Organization Corporate Statistical Database) data, Italy is the fifth largest European goat cheese producer recording an amount of 4500 tons produced in 2014 placing them after France, Greece, Spain, and Bulgaria [7]. Lombardy is the second-largest Italian region for goat livestock number, with 98,766 female goats bred out of the total 827,418 in Italy (12%) [8]. Half of the goat farms in Lombardy have small herds, from 20 to 60 lactating goats, represented 95% by Saanen or Alpine breeds [9]. Of overall Italian goat milk production (43,444 tons), Lombardy contributes 16% (7076 tons) [10], being second only to Sardinia, with an average production of 549 ± 216 kg of milk per goat per lactation [9]. The caprine livestock production systems adopted in dairy goat farms in Italy are very diversified, ranging from intensive, indoor, highly productive, and specialized productions, to extensive outdoor systems, with traditional local breeds and with seasonal productions [11,12]. As evidenced in a study conducted on 173 dairy goat farms in Lombardy, in almost 30% of the studied farms, goats had access to pasture during the spring and summer season, with advantages in terms of reducing feeding costs [12].

In Italian small goat farms located in the mountains, the conventional production system can be considered a “low-input system” (LIS), characterized by feeding practices similar to those used in organic farming, but not complying with all the restrictions established by organic standards [13]. The LIS is known to be strictly related to milk quality in ruminants [14] and, specifically, in goats [15,16], mainly due to the access to pasture. Basically, LIS goats are forage-fed plus given concentrate supplementation mostly during a certain lactation period and physiological stages [2], whereas in the conventional “high-input system” (HIS) goats are fed diets richer in grain and characterized by a lower forage/concentrate (F/C) ratio. As a matter of fact, the concentrates and forage intake and the nature of individual foodstuffs have an impact on the characteristics of goat milk and milk products [17]. Even if small mountain farms are not always organic-certified and do not always practice pasture, the yielded milk has shown to have a similar nutritive and functional quality to its organic counterpart. The main differences between the LIS and the HIS are recorded in the milk production and content of nutritionally favorable fatty acids, such as n3-series PUFA (polyunsaturated fatty acids), CLA, and branched-chain fatty acids (BCFA) [18,19,20,21,22]. Moreover, small mountain farms are usually associated with an on-farm cheese making process and the sale of the products directly on the farm or in local shops, comprising a market segment that is generally related to high-quality and traditional products, strictly linked to territory and seasonality [4]. Typically, Goat cheese produced and sold in Italy are small-sized and soft, obtained by lactic or rennet coagulation. Fresh cheese obtained by a combination of a slow acidification through the addition of mesophilic starter culture combined with heat [23] is characterized by 50–65% moisture, a lipid content up to 15–18 g/100 g and a protein content up to 16–29 g/100 g [24]. Hard and semi-hard goat cheese, represented by the presamic curd obtained by the enzymatic action of kid rennet on casein micelle [25], is characterized by 35–45% moisture, a lipid content up to 24–32 g/100 g and a protein content up to 20–26 g/100 g [24].

The on-farm cheese making appears to be fundamental since farmers can also benefit from the added value of the processing and sale, making the business more profitable [26]. Finally, LIS are often associated by consumers to the idea of high welfare levels, because outdoor and extensive farming systems allow animals to behave in a more natural way [27]. In goats, a study using qualitative behavior analysis (QBA) highlighted a better emotional state in animals on pasture that in animals housed indoors [28].

Food products can also be described through descriptive factors that contribute to the definition of total quality. These factors can be divided into either intrinsic to the food itself, as chemical composition, aroma, and nutritional properties, or those that are extrinsic. Extrinsic attributes are aspects related to the product but not physically a part of it. They are linked to natural and cultural assets and are hidden into a broader food quality concept. In the case of animal origin products, examples of important extrinsic quality attributes are environmental sustainability and animal welfare, which may be related to the production process and to the place of origin.

To date, scientific studies about dairy goat products are fewer if compared to those regarding raw goat milk. The present study aimed to show the potential of the different production systems to affect some intrinsic (fatty acid composition of goat cheese) and extrinsic (animal welfare) quality attributes of goat products on three farms in Lombardy.

## 2. Materials and Methods

### 2.1. Animals, Feeding and Housing Practices

Three farms from Lombardy were involved in this study, conducted from March (4 ± 1 weeks of lactation) to October 2017, covering three quarters of the milking season. The first farm (342 m above sea level, a.s.l.) was represented by an organic-certified farm [29], breeding 45 Alpine lactating goats. On this organic farm (O), goats had access to pasture for 224 days/year and 4 h/day after the morning milking. Goats were rationally grazed with mixed vegetative forages during the entire sampling season and the access to fresh grass at pasture was controlled. In the barn, goats received forages, consisting of alfalfa hay (1000 g/d per animal) and polyphite grass hay (500 g/d per animal) distributed twice a day, and a mixture of organically-certified maize/barley (3/1) grains, supplied twice a day during milking (800 g/d per animal). The second farm (M) was represented by a typical mountain farm (980 m a.s.l.), with 39 Alpine goats, where the production system can be considered a LIS, since goats were reared and fed primarily in the barn but a diet with a high F/C ratio and receiving local fresh grass when available on the farm. In farm M, feed supplements were provided to goats, including polyphite grass hay (first harvest) or fresh grass, when available, offered ad libitum plus alfalfa hay (500 g/d per animal) distributed during the morning milking and a commercial mixture of concentrates (1000 g/d per animal) distributed twice a day, during milking. Finally, the third farm (C) was represented by a conventional-lowland dairy goat farm (278 m a.s.l.) with 42 Saanen lactating goats, permanently reared in the barn. The diet included local ryegrass hay offered ad libitum, alfalfa hay offered once a day (500 g/d per animal) during the first part of lactation, and commercial feed, represented by a mixture of flaked and flaked cereals and flour, provided twice a day (1200 g/d per animal) during milking. In all three farms, goats had free access to water and to salt integrators. The F/C ratio for each farm was calculated on the basis of the average daily feed intake and it is reported in Table 1. Average feed intake was estimated by mean of dry matter intake (DMI) prediction models for lactating goats as reported by Pulina et al. [30], taking into account average goat size and the average milk yield during the sampling season of the study. Dry matter of feedstuffs was calculated by fodder analysis according to official methods [31].

Cheese samples consisted of two different dairy goat products typically produced and sold in Italy. The former was represented by fresh cheese samples, processed by lactic coagulation (about 120 g of weight), collected once a month from March to October in each farm, except for farm M that was lacking of one sample, for a total amount of 23 samples (8 in O, 7 in M, 8 in C). The latter was represented by semi-hard cheese blocks, processed by rennet coagulation (about 100 g of weight), obtained after 3 ± 1 weeks of ripening and collected once a month from April to October in each farm, with the exception of O farm in which one sample was lacking, for a total amount of 20 samples (6 in O, 7 in M, 7 in C). Both fresh and semi-hard cheese samples (a total amount of 43 samples) were stored at −20 °C until fatty acids analysis was performed.

### 2.2. Fatty Acids Analysis

Fat was extracted from cheese samples by the Folch method [32], using a mixture of chloroform/methanol (2:1). After lipid quantification, an aliquot corresponding to 40–50 mg of fat was used for fatty acids analysis by mean of gas-chromatography (GC) and flame ionization detection (FID). In order to perform the GC-FID analysis, fatty acids were methyl-esterified by mean of base-catalysed methanolysis of glycerides, following the method of Christie [33]. Briefly, lipids were dissolved in 1 mL of diethyl ether, then 50 μL of methyl acetate and 100 μL of sodium methoxide in methanol 1M were added. After 5 min at room temperature, the reaction was stopped by adding 50 μL of a saturated solution of oxalic acid in diethyl ether. The solution was centrifuged at 1500× *g* for 5 min, then 200 μL of the supernatant was taken and immediately injected in the GC system. The GC system was represented by an Agilent gas-chromatograph model 6890, fitted with an automatic sampler model 7683 and a FID detector (Agilent Technologies, Santa Clara, CA, USA). Chromatographic conditions were set following the method described in Lopez et al. [22]. Individual fatty acids methyl esters were identified by comparing sample peak retention times with standard mixtures and pure standard methyl esters from Sigma Aldrich (Saint Louis, MO, USA) and then were expressed as percentage of total fatty acids. A gas-chromatographic correction factor was applied in order to take into account the lower response of the FID for compounds represented by a lower number of carbon atoms (4:0, 6:0, 8:0, 10:0, and 12:0) [34].

### 2.3. Welfare Assessment

Animal welfare was evaluated using the 1st-level AWIN (Animal Welfare Indicators) welfare assessment protocol for goats [35,36]. In agreement with the AWIN protocol, in each farm the evaluation was carried out in a pen selected as having the highest risk for animal welfare, according to the following criteria: highest density, lower feeding space/animal ratio, lower drinking place/animal ratio, and presence of both horned and hornless goats in the same pen. Therefore, if more than one pen was present in a farm, the number of assessed goats per farm does not correspond to the total number of lactating goats, but to that of goats in the selected pen (number of goats assessed per farm: O = 36; M = 24; C = 39). The assessment included the collection of the following indicators when the animals were at the feed rack after the morning milking: incorrect disbudding, presence of abscesses, kneeling at the feed rack, queueing at feeding, queueing at drinking, hair coat conditions, oblivion (isolated goats), thermal stress (either heat or cold stress signs). After the collection of these indicators, the goats were observed from outside the pen for 10 min for the qualitative behavior assessment (QBA; [37]). Then the assessor entered the pen to evaluate the quality of the human-animal relationship, performing the latency to first contact test, which consists in measuring the time until the first goat spontaneously gets in touch with the operator after his/her entrance into the pen [38]. Finally, the bedding quantity (sufficient, insufficient) and cleanliness (clean, dirty and/or wet) were assessed, as an indirect index of animal comfort, and the number of goats that presented severe signs of lameness was recorded [36].

### 2.4. Statistical Analysis

The difference among the three farms (O, M, C) for fatty acid analysis was evaluated by mean of the analysis of variance. Normal distribution (Shapiro–Wilk test) and homogeneity of variances (Levene test) were confirmed and comparison among means was performed by the ANOVA test and the Welch ANOVA Ftest. The Tukey-HSD test was used as the post-Hoc test for comparison of the means among different farms. Significance was declared at *p* ≤ 0.05 (*) and *p* ≤ 0.01 (**). Afterwards, a multivariate analysis was performed by mean of a combined principal component analysis (PCA) and linear discriminant analysis (LDA) approach. In a first step, PCA was performed in order to reduce the dimensionality of the final data matrix. The PCA was performed including milk samples obtained in a previous study [22] analyzing the bulk milk used to produce cheese samples analyzed in the present work. Variables were selected when PC loadings score were > |0.5|. In a second step, LDA was performed in a new matrix, including the variables selected by PCA. Milk samples were included in the model as training set and cheese samples as validation set, in order to verify if the discrimination among samples coming from different farms was satisfying by means of the multivariate approach chosen.

Animal welfare indicators were expressed as the proportion of animals that do not present a specific welfare problem out of the number of animals observed, except for the latency to first contact, which was expressed in seconds, and the QBA, which was expressed as scores on a visual analogue scale and then submitted to PCA. Statistical analysis was performed using JMP Pro 14.0.0 (SAS Institute Inc., Cary, NC, USA).

### 2.5. Ethical Approval

This research did not involve any experimental practice performed on living animals by the authors and no biological matter was collected. Only cheese samples were collected, after the on-farm production stage. Authors guarantee that in the three farms involved in the present study, all the applicable guidelines for animal welfare established by the harmonized EU rules were followed. No approval by the ethics committee of authors’ institution (University of Milan) was requested.

## 3. Results and Discussion

Results obtained by the fatty acid (FA) analysis on fresh and ripened cheese samples are reported in Table 1. 

Dairy goat products are generally enriched in the content of short-chain saturated fatty acids (sc-SFA) 6:0, 8:0, and 10:0, commonly known as caproic, caprylic, and capric acid, considered responsible for the characteristic flavor of goat products [42]. SFA are known to be directly related to obesity, cardiovascular, and metabolic diseases in humans [43], caproic, caprylic, and capric acid have been recognized to have a unique metabolic ability in humans, since they provide direct energy instead of being deposited in the adipose tissue and lower serum cholesterol. For this reason, they have already achieved an important role in the functional evaluation of goat products in human nutrition and medicine [1]. No significant differences were found in the content of such fatty acids among the three farms involved in this study, with the exception of capric acid that was higher in semi-hard cheese samples collected in farm C (9.82%) than in samples collected in farm M (8.37%). Short- and medium-chain FA are synthesized in the mammary gland mainly by FA synthase, starting from volatile fatty acids produced by rumen activity as precursors. Moreover, mammary delta-9-desaturase can also convert stearic acid (18:0) to oleic acid (18:1 n9). Stearic acid and oleic acid in dairy products could be derived from the feed intake, the body fat mobilization, or by the hydrogenation processes that occur in the rumen [44]. Oleic acid, particularly, is known to have positive effects on human health, mainly toward the digestive and cardiovascular systems and the inflammatory response, and it seems to play a pivotal role in the management of the oxidative stress and in the development of the nervous system [45]. Thus, in dairy products, it is considered advisable to keep the oleic acid/stearic acid proportion as high as possible, in order to improve the nutritional characteristics of milk and related products [44]. The amount of oleic acid in caprine products is strongly influenced by the diet, since (i) it is inversely related to the amount of concentrates supplied to goats [46] and (ii) grazing practice increases oleic acid concentration in milk, through the hydrogenation and desaturation processes against PUFA present in the pasture [44]. In agreement with these findings, we found the lowest concentration of oleic acid in cheese samples coming from farm C (17.78–18.09%), where goats were fed with the highest amount of concentrates. Conversely, the highest concentrations were found in samples from farm O (19.70–19.74%) and M (21.11–21.93%), where goats consumed less concentrate and more fresh grass or pasture browsing plants. PUFA biohydrogenation in the rumen also leads to the formation of many intermediate products, such as trans (t9 + t11) 18:1 isomers [47]. It has been demonstrated that the consumption of trans fatty acids, in contrast to cis fatty acids, can lead to pleiotropic negative effect on human health, including pro-inflammatory activity, adverse lipid effects, endothelial dysfunctions, insulin resistance, etc. [48]. At the same time, recent scientific opinions state that the consumption of trans FA by mean of ruminant products in actual diets is very modest and that, at such levels, they do not appear to be major contributors to health risk [48]. In our work, we found a significantly higher content of elaidic acid (t9, 18:1) in samples from farm C (0.50%), followed by farm M (0.39–0.41%) and then by farm O (0.31%). No significant differences were found in the three farms regarding the content of vaccenic acid (t11, 18:1) that is the main trans 18:1 isomer in dairy products. These results are in accordance with the scientific evidence stating that the concentrates proportion in goat diets is positively related to the content of the trans 18:1 isomers in milk, with the exception of vaccenic acid [49].

PUFA are not synthesized in ruminants tissues, thus their concentration in milk and dairy products depend on the amount of PUFA provided to animals by the diet that partially escapes from the rumen microbial hydrogenation activity [44]. It is known that fresh forage is richer in PUFA if compared with hay [50,51,52]. Although we would have expected to find higher amounts of PUFA in samples collected in the farms where goats were led to pasture, we detected a higher total amount of PUFA in farm M (4.28–4.44%) and C (4.43–4.62%) rather than in farm O (3.69–3.77%). As a matter of fact, the higher amount of PUFA in both fresh and semi-hard cheese samples collected in farm C can be mainly imputed to the significantly higher content of linoleic acid (c9c12, 18:2) found in such samples (3.17–3.28% in farm C vs. 2.13–2.14% in farm O and 2.47–2.71% in farm M). It is known that linoleic acid reaches significantly higher amounts in dairy products when the diet provided to goats contains a higher proportion of concentrates [53]. Actually, the diet supplied to goats in farm C was characterized by the lowest F/C ratio and the higher amount of concentrates, naturally rich in linoleic acid [52]. At the same time, alpha-linolenic acid (18:3 n3) followed the opposite trend, resulting in higher concentrations in samples collected in farm O (0.72–0.78%) and M (0.76–0.82%) than in farm C (0.43–0.45%). It is known that alpha-linoleic acid content is strongly influenced by the consumption of fresh grass and natural grassland hay, since they are natural sources of this FA, and a positive correlation between the content of alpha-linolenic acid in the diet and in dairy products exists [44,53]. In agreement with this, we found the highest alpha-linoleic content in cheese samples collected in farms where goats’ diet was represented by higher pasture and fresh grass intake and lower amounts of concentrates were supplied than in the conventional intensive farm (thus, a lower F/C ratio). These results are in accordance with the outcomes of other previous studies performed on organic versus conventional goat milk [15,22]. In addition to total PUFA and alpha-linoleic acid, also linoleic acid conjugated isomers (CLA), mainly represented by c9t11 18:2, are considered as markers of fiber intake in ruminants, showing higher percentages when goats are fed with fresh grass [16,53]. Several studies investigated CLA content in goat milk and cheese. The outcomes of these studies suggested that goat products include CLA reaching amounts < 1% of total FA, depending on several factors (lactating period, genetics, dietary factors, etc.). The amount of CLA found in goat dairy products are comparable to those found in bovine dairy products and lower than those found in sheep dairy products [54,55]. Particularly in their papers, Tsiplakou et al. [15,56] found that no differences were detectable when analyzing goat products obtained by organic vs. conventional production system or outdoor vs. indoor farming system, even suggesting that the ruminant species is the primary factor influencing CLA content in dairy products. Unfortunately, the analytical equipment used in the present study, provided with a 60 m length column, did not allow the separation of c9t11 18:2 that coeluted with 20:0 peak.

Linoleic acid and alpha-linoleic acid in milk and dairy products are the most representative FA for the n6- and n3- series, respectively. It is well known that n6- and n3- series FA are essential fatty acids (EFA) for humans, with peculiar and not inconvertible functional roles. n6- and n3- FA generally show opposite physiological functions toward cardiovascular diseases, cancer, inflammatory and autoimmune diseases and neural development, thus their balance is considered fundamental in order to assure their biochemical efficiency [57]. It has been estimated that, nowadays, common diets can reach an n6/n3 ratio ranging from 10:1 to 20:1, whereas the target ratio, suitable to obtain a functional activity toward the prevention of the above mentioned diseases, should be from 1:1 to 2:1 maximum [57]. In the past, some international nutritional organizations have recommended an advisable n6/n3 ratio in human diet ranging between 5:1 and 10:1, as resumed by Ma et al. [58] in a review. In our study, we found an n6/n3 ratio significantly higher in samples collected in farm C in both the typologies of products analyzed, reaching values > 6:1. On the contrary, in farm O and M, the n6/n3 ratio reached values ranging from 2.59:1 to 3.09:1, closer to the suggested optimum of 1:1. This result is highly significant, since samples coming from the conventional lowland production system (farm C) showed values for this ratio two times higher than the values recorded in samples collected in the two low-input system farms (O and M). This suggested that the organic and the mountain production systems led to the production of goat cheeses characterized by a more balanced EFA profile.

Many odd and branched chain fatty acids (OBCFA) were identified in fresh and semi-hard cheese samples in this study. OBCFA are characteristic FA of ruminants products, since they are components of rumen bacteria membranes that partially are absorbed and included in ruminants fats [59,60]. In our study, the sum of total OBCFA resulted higher in the two LIS farms (4.62–4.66% in farm O and 4.43–4.50% in farm M) than in the HIS farm (3.91–4.00% in farm C). Previously, other authors found a positive correlation between the F/C ratio in the diet supplied to goats and the OBCFA content in yielded milk [22,46], particularly the iso forms of the branched chain FA (BCFA). It is known that rumen cellulolytic microbial populations are enriched in iso FA whereas amylolytic bacteria are enriched in the anteiso form and in linear odd-chain FA (OCFA) [59,60]. It has been demonstrated that many dietary factors, including the F/C ratio, can lead to modification of the rumen function and of the microbial populations, so that diets rich in starch reduce iso FA and, on the contrary, diets rich in forages increase iso FA in milk fat [60]. According to this, we found iso 14:0 and iso 16:0 amounts significantly higher in samples coming from farm O and M than in samples from farm C, in both fresh and semi-hard cheese. These results supported the results of other authors [61,62] who found a positive correlation between the fiber content in the diet and iso 14:0 amount in goat milk, suggesting an enrichment of the cellulolytic populations in goats’ rumen when fed with higher quantity of fiber. OBCFAs, particularly the branched-chained ones, are known to be important functional components in human nutrition since they showed (i) a positive activity on the gastrointestinal health, (ii) a cytotoxic activity comparable with the one of conjugated linoleic acid and (iii) positive effects against cardiovascular problems, such as atherosclerosis, thrombogenesis, coronary heart disease, blood cholesterol levels regulation and so on [63,64,65,66]. Moreover, as evidenced by Haenlein [1], goat milk generally contains a higher number of minor branched chain FA that, together with the lower content of trans 18:1 FA, represent a benefit for coronary heart disease risk.

Finally, we calculated the health lipid indices AI (atherogenic index), TI (thrombogenic index) and h/H (hypo/Hypercholesterolemic Index) that, together with n6/n3 ratio, are generally used to evaluate the nutritional value of dairy fat [67]. Our data for AI and TI were comparable to those previously obtained by other authors for goat cheese [54,68,69]. Rafiee-Yarandi et al. [70] suggested that dairy products characterized by lower AI and TI values have a little incidence on the development of atherosclerosis and thrombosis in humans. In our study, we recorded the most interesting results in semi-hard cheese samples, where the AI and TI were significantly lower in farm M (2.25 and 2.52, respectively) than in farm O (2.84 and 2.84) and C (3.11 and 3.32). According to Hanus et al. [67] and Sant’Ana et al. [69] we found the highest values for AI and TI in samples collected in the farm managed with a high-input production system (more concentrates supplied, no pasture) and lower values for the farms managed by the low-input production systems (higher fiber content in the diet, access to the pasture). On the contrary, we found the highest h/H value in semi-hard cheese samples collected in farm M (0.79), followed by samples from farm O (0.64) and C (0.57), in a range comparable to the one previously detected in goat milk [71]. It has been suggested that dairy products characterized by a high h/H ratio potentially have a protective effect against cardiovascular diseases [70].

**Figure 1:**
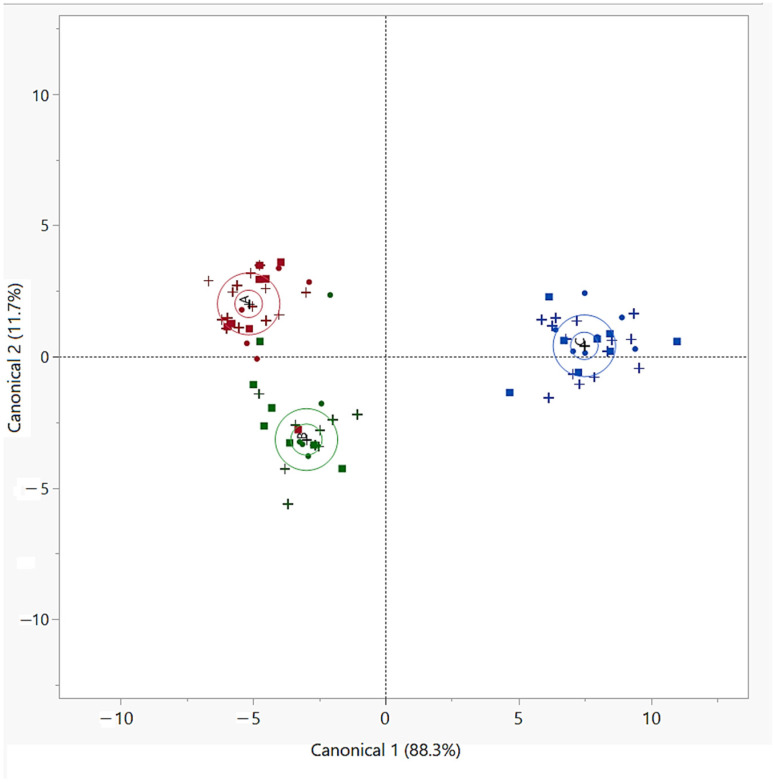
Canonical plot of the linear discriminant analysis performed on cheese samples, grouped by farm (O, M, C), using milk samples from Lopez et al. [22] as training set. In the plot, each group is associated with a 95% confidence ellipse for the mean and a 50% prediction ellipsoid. Legend: red = Farm O (organic-certified farm); green = Farm M (mountain farm); blue = Farm C (conventional lowland farm); + = milk; ■ = fresh cheese; ● = semi-hard cheese.

In order to define if a discrimination among samples from the three farms was feasible by means of FA analysis, we applied a multivariate approach. A linear discriminant analysis (LDA) was performed with twenty-one variables as covariates, represented by OBCFA, n3- and n6- series PUFA, elaidic acid, 22:0, 20:1 n9, AI and TI. Such variables were selected in a first step, in which principal component analysis (PCA) was performed on data matrix to reduce dataset dimensionality, choosing factors associated with loadings > |0.5|. In Figure 1, the canonical plot obtained after the development of the LDA on our dataset, using the corresponding milk samples analyzed in a previous work [22] as training set, is shown.

In the canonical plot, it is clear that almost all samples analyzed in this study were assigned to the group they belonged to, with 3 samples over the 17 of the validation set being misclassified (misclassification rate: 17.64%). Particularly, two samples collected on farm M were misclassified as samples of the O group and one sample collected on farm O was misclassified as a sample of the M group, whereas all samples from farm C were correctly classified, showing a clear differentiation from the samples collected in the other two farms.

In Figure 1, the similarity among O and M samples is clear. This phenomenon reflected the similarity observed in fatty acids percentages in O and M groups and it suggests that the difference in the production method (LIS versus HIS) was the main factor affecting the discrimination between O and M vs. C cheese samples. This result confirmed the assumption that farms where goats were fed with fresh grass or were allowed to graze (the organic-certified and the mountain farm) produced cheese represented by a FA composition clearly distinguishable from cheese coming from the conventional-lowland farm, where more concentrates were supplied to goats. Particularly, in the farms O and M, the higher F/C ratio (70:30 and 60:40, respectively) and the consumption of fresh grass and browsing plants, led to higher contents of health promoting fatty compounds, as previously remarked by other authors [2,72,73,74].

As to welfare outcomes deriving from the application of the AWIN welfare assessment protocol for goats, farm O presented the highest proportion of animals with no welfare problems and farm C also showed a good welfare level, whereas farm M showed the most critical situation, especially due to the high proportion of goats with poor hair coat conditions and presenting kneeling at the feeding rack, probably due to a poor management (Table 2).

In farm O, as well as in farm M, we can also observe a very good human-animal relationship, as shown by the very low time of latency to the first contact test (2 s in farm O and 1 s in farm M), whereas in farm C goats approached the assessor only after 162 s. Bedding was dirt in all the three farms, and its quantity was also insufficient in farm M.

**Figure 2:**
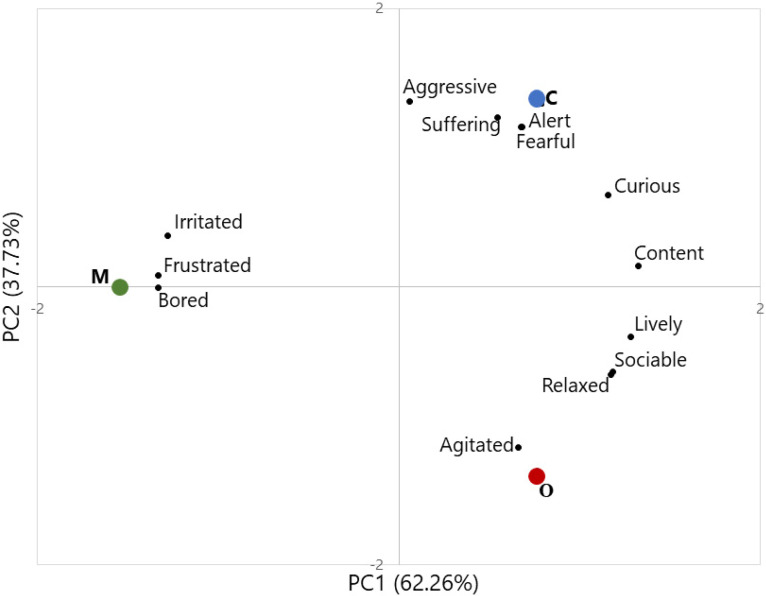
Scores plot of the principal component analysis performed on using QBA (qualitative behavior analysis) results. Legend: red = farm O (organic-certified farm); green = farm M (mountain farm); blue = farm C (conventional lowland farm); black = descriptors.

These welfare conditions are confirmed by QBA results, presented in Figure 2. PCA plot shows that farm M is positioned on the left side of PC1 (62.26% of explained variance), which carries information about the valence of the emotional state of goats. In fact, this farm was mainly characterized by descriptors such as irritated, frustrated and bored, indicative of a negative emotional state. On the contrary, farms O and C are on the right side of PC1, with higher loadings of positive descriptors such as content, lively, sociable, curious and relaxed. Some differences between farms O and C can be observed on PC2 (37.73% of explained variance), where C is characterized by higher loadings of aggressive, alert, suffering and fearful, and O is mainly described as agitated.

Overall, welfare outcomes showed a higher level of welfare in farm O, confirming the attention paid by organic producers to animal welfare, which is considered a priority in organic livestock farming by the current European legislation [75]. However, high levels of welfare can be achieved also in conventional farms, as highlighted by our results for farm C. This suggests that no clear relationship can be observed between the level of animal welfare and fatty acid composition of goat cheese.

## 4. Conclusions

Results obtained in this study confirmed the conclusions proposed in previous works on the fatty acid composition of goat milk. Generally, cheese from the LIS farms showed a more favorable fatty acid composition and better values for the health lipid indices, supporting the potential assessment of these products to the “functional food” market. Concerning origin discrimination among cheese from the three farms, a partial over imposition among samples coming from the two low-input management systems was evidenced, with a clear discrimination from cheese samples collected in the high-input system farm. However, we observed that, although LIS farms seem to provide cheese with better intrinsic qualities, they are not always able to guarantee high extrinsic qualities, for example in terms of animal welfare, as demonstrated by the contrasting results about lipid quality of cheese and goats’ welfare in farms M and C.

As modern consumers are interested in both intrinsic and extrinsic quality of animal products our results suggest that assurance schemes cannot contain only information on the livestock system, because this information alone is not enough to provide a complete view of product quality; a more complete and detailed information on different quality traits should be provided in order to increase consumers’ awareness and to allow them to make a more conscious food consumption.

## Figures and Tables

**Table 1:** Fatty acids composition of fresh and semi-hard goat cheese samples. Data are expressed as g/100 g of total fatty acids (mean ± standard deviation).

	Fresh Cheese					Semi-Hard Cheese				
Farm	O	M		C		O	M	C		
F/C ^1^	70/30	60/40		50/50		70/30	60/40	50/50		
n	8	7		8		6	7	7		
Saturated fatty acids (SFA)					sign					sign
4:0	1.98 ± 0.22	1.87 ± 0.25		1.99 ± 0.17		2.01 ± 0.25 ^ab^	2.42 ± 0.41 ^a^	1.93 ± 0.10 ^b^		*
6:0	1.98 ± 0.21	1.88 ± 0.15		2.05 ± 0.18		1.98 ± 0.20	2.15 ± 0.37	1.96 ± 0.08		
8:0	2.63 ± 0.28	2.12 ± 0.93		2.76 ± 0.24		2.58 ± 0.23	2.01 ± 1.29	2.62 ± 0.08		
10:0	9.52 ± 0.85	9.05 ± 1.05		10.04 ± 0.65		9.41 ± 0.47 ^ab^	8.37 ± 2.52 ^b^	9.82 ± 0.38 ^a^		*
12:0	5.43 ± 1.71	5.65 ± 2.19		5.19 ± 0.70		5.08 ± 0.89	4.08 ± 0.70	5.31 ± 0.78		
14:0	11.18 ± 2.12	11.20 ± 2.27		11.20 ± 0.59		10.91 ± 1.53 ^ab^	9.85 ± 0.70 ^b^	11.63 ± 0.73 ^a^		*
16:0	25.96 ± 2.34 ^ab^	23.80 ± 1.49 ^a^		26.95 ± 1.58 ^b^	*	26.25 ± 1.91 ^a^	23.68 ± 1.99 ^b^	27.30 ± 0.93 ^a^		**
18:0	9.16 ± 3.03	9.97 ± 3.49		8.96 ± 1.44		9.37 ± 2.33 ^ab^	11.92 ± 0.71 ^a^	8.89 ± 1.62 ^b^		*
20:0 ^2^	0.84 ± 0.18	1.01 ± 0.15		0.86 ± 0.10		0.93 ± 0.09	0.95 ± 0.37	0.88 ± 0.07		
22:0	0.10 ± 0.03 ^ab^	0.12 ± 0.04 ^a^		0.07 ± 0.02 ^b^	**	0.11 ± 0.02	0.08 ± 0.05	0.07 ± 0.01		
ΣSFA	70.88 ± 3.04	68.74 ± 3.24		71.77 ± 1.02		70.75 ± 1.66 ^a^	67.58 ± 2.63 ^b^	72.16 ± 1.01 ^a^		**
Monounsaturated fatty acids (MUFA)										
14:1	0.26 ± 0.22	0.27 ± 0.18		0.19 ± 0.07		0.22 ± 0.13	0.20 ± 0.29	0.18 ± 0.07		
16:1	0.77 ± 0.33	0.71 ± 0.21		0.60 ± 0.11		0.71 ± 0.17	0.64 ± 0.27	0.59 ± 0.14		
t9, 18:1	0.31 ± 0.07 ^a^	0.39 ± 0.04 ^b^		0.50 ± 0.06 ^c^	**	0.31 ± 0.04 ^a^	0.41 ± 0.12 ^ab^	0.50 ± 0.04 ^b^		**
t11, 18:1	1.06 ± 0.39	1.31 ± 0.09		1.32 ± 0.21		1.17 ± 0.32	1.60 ± 0.73	1.41 ± 0.45		
c9, 18:1	19.70 ± 2.62	21.11 ± 3.22		18.09 ± 0.71		19.74 ± 1.25 ^a^	21.93 ± 1.79 ^b^	17.78 ± 0.68 ^c^		**
c11, 18:1	0.41 ± 0.05	0.38 ± 0.06		0.40 ± 0.02		0.41 ± 0.05 ^ab^	0.47 ± 0.06 ^a^	0.40 ± 0.02 ^b^		*
20:1n9	0.05 ± 0.00 ^a^	0.04 ± 0.01 ^a^		0.07 ± 0.01 ^b^	**	0.05 ± 0.01 ^a^	0.04 ± 0.02 ^a^	0.07 ± 0.01 ^b^		**
ΣMUFA	22.85 ± 2.60 ^ab^	24.51 ± 2.88 ^a^		21.33 ± 0.77 ^b^	*	22.89 ± 1.39 ^a^	25.59 ± 2.49 ^b^	21.09 ± 0.83 ^a^		**
Polyunsaturated fatty acids (PUFA)										
t9t12, 18:2n6	0.21 ± 0.07	0.26 ± 0.02		0.25 ± 0.04		0.23 ± 0.06	0.26 ± 0.08	0.24 ± 0.04		
c9t12, 18:2n6	0.12 ± 0.02 ^a^	0.17 ± 0.06 ^b^		0.15 ± 0.03 ^ab^	*	0.11 ± 0.01	0.11 ± 0.07	0.13 ± 0.02		
c9c12, 18:2n6	2.13 ± 0.41 ^a^	2.47 ± 0.43 ^a^		3.28 ± 0.29 ^b^	**	2.14 ± 0.28 ^a^	2.71 ± 0.12 ^b^	3.17 ± 0.12 ^c^		**
18:3n6	0.02 ± 0.02	0.03 ± 0.02		0.03 ± 0.01		0.02 ± 0.02	0.03 ± 0.02	0.03 ± 0.02		
18:3n3	0.72 ± 0.13 ^a^	0.76 ± 0.07 ^a^		0.45 ± 0.13 ^b^	**	0.78 ± 0.09 ^a^	0.82 ± 0.13 ^a^	0.43 ± 0.10 ^b^		**
20:2n6	0.05 ± 0.02	0.04 ± 0.01		0.04 ± 0.02		0.03 ± 0.02	0.02 ± 0.02	0.03 ± 0.00		
20:3n6	0.03 ± 0.02	0.03 ± 0.00		0.03 ± 0.02		0.02 ± 0.02	0.03 ± 0.03	0.03 ± 0.01		
20:4n6	0.16 ± 0.02 ^a^	0.18 ± 0.02 ^ab^		0.19 ± 0.03 ^b^	*	0.15 ± 0.01 ^a^	0.19 ± 0.03 ^b^	0.18 ± 0.01 ^b^		**
20:5n3	0.09 ± 0.02	0.14 ± 0.11		0.07 ± 0.02		0.09 ± 0.03	0.07 ± 0.04	0.06 ± 0.03		
22:5n3	0.17 ± 0.02 ^ab^	0.16 ± 0.04 ^a^		0.13 ± 0.04 ^b^	**	0.19 ± 0.03 ^a^	0.20 ± 0.05 ^a^	0.12 ± 0.04 ^b^		**
ΣPUFA	3.69 ± 0.61 ^a^	4.28 ± 0.35 ^ab^		4.62 ± 0.37 ^b^	**	3.77 ± 0.40 ^a^	4.44 ± 0.31 ^b^	4.43 ± 0.25 ^b^		**
Σn3	0.98 ± 0.14 ^a^	1.10 ± 0.13 ^a^		0.64 ± 0.12 ^b^	**	1.06 ± 0.13 ^a^	1.10 ± 0.18 ^a^	0.61 ± 0.15 ^b^		**
Σn6	2.72 ± 0.51 ^a^	3.18 ± 0.40 ^a^		3.98 ± 0.33 ^b^	**	2.71 ± 0.36 ^a^	3.34 ± 0.20 ^b^	3.81 ± 0.15 ^c^		**
n6/n3	2.79 ± 0.47 ^a^	2.95 ± 0.63 ^a^		6.38 ± 1.23 ^b^	**	2.59 ± 0.42 ^a^	3.09 ± 0.42 ^a^	6.53 ± 1.39 ^b^		**
Odd and branched chain fatty acids (OBCFA)										
11:0		0.12 ± 0.05		0.13 ± 0.07	0.10 ± 0.02		0.12 ± 0.03 ^a^	0.06 ± 0.04 ^b^	0.10 ± 0.03 ^ab^	*
13:0		0.12 ± 0.03		0.12 ± 0.04	0.10 ± 0.01		0.12 ± 0.02	0.07 ± 0.04	0.10 ± 0.02	
*iso* 14:0		0.15 ± 0.03 ^a^		0.13 ± 0.03 ^a^	0.10 ± 0.01 ^b^	**	0.16 ± 0.01 ^a^	0.14 ± 0.02 ^a^	0.10 ± 0.01 ^b^	**
*iso* 15		0.23 ± 0.03		0.24 ± 0.05	0.23 ± 0.03		0.24 ± 0.00	0.26 ± 0.04	0.24 ± 0.03	
*anteiso* 15:0		0.65 ± 0.09 ^a^		0.65 ± 0.05 ^a^	0.52 ± 0.05 ^b^	**	0.65 ± 0.06 ^a^	0.57 ± 0.08 ^ab^	0.54 ± 0.04 ^b^	*
15:0		1.13 ± 0.10 ^a^		1.10 ± 0.08 ^a^	0.90 ± 0.05 ^b^	**	1.15 ± 0.07 ^a^	1.07 ± 0.06 ^a^	0.94 ± 0.04 ^b^	**
*iso* 16:0		0.33 ± 0.06 ^a^		0.27 ± 0.07 ^ab^	0.25 ± 0.03 ^b^	*	0.35 ± 0.04 ^a^	0.32 ± 0.05 ^a^	0.25 ± 0.03 ^b^	**
*iso* 17:0		0.35 ± 0.04		0.36 ± 0.06	0.40 ± 0.03		0.36 ± 0.03	0.39 ± 0.02	0.40 ± 0.03	
*anteiso* 17:0		0.82 ± 0.21		0.79 ± 0.10	0.72 ± 0.07		0.79 ± 0.13	0.68 ± 0.04	0.72 ± 0.07	
17:0		0.72 ± 0.13		0.73 ± 0.19	0.60 ± 0.06		0.74 ± 0.09 ^a^	0.86 ± 0.07 ^a^	0.60 ± 0.05 ^b^	**
17:1		0.33 ± 0.03 ^a^		0.33 ± 0.02 ^a^	0.23 ± 0.02 ^b^	**	0.33 ± 0.03 ^a^	0.34 ± 0.03 ^a^	0.23 ± 0.01 ^b^	**
ΣOCFA		2.10 ± 0.16 ^a^		2.07 ± 0.16 ^a^	1.70 ± 0.06 ^b^	**	2.54 ± 0.16 ^a^	2.36 ± 0.23 ^ab^	2.25 ± 0.10 ^b^	*
ΣBCFA		2.52 ± 0.22 ^a^		2.43 ± 0.13 ^a^	2.21 ± 0.11 ^b^	**	2.13 ± 0.05 ^a^	2.07 ± 0.11 ^a^	1.75 ± 0.05 ^b^	**
ΣOBCFA		4.62 ± 0.26 ^a^		4.50 ± 0.27 ^a^	3.91 ± 0.14 ^b^	**	4.66 ± 0.16 ^a^	4.43 ± 0.27 ^a^	4.00 ± 0.15 ^b^	**
Health Lipid Indices										
AI ^3^	2.95 ± 0.84	2.64 ± 0.73	2.97 ± 0.2		2.84 ± 0.48 ^a^	2.25 ± 0.27 ^b^	3.11 ± 0.30 ^a^			**
TI ^4^	2.92 ± 0.44 ^ab^	2.59 ± 0.22 ^a^	3.20 ± 0.23 ^b^	**	2.84 ± 0.22 ^a^	2.52 ± 0.20 ^b^	3.32 ± 0.19 ^c^			**
h/H ^5^	0.65 ± 0.15	0.74 ± 0.18	0.60 ± 0.05		0.64 ± 0.10 ^a^	0.79 ± 0.09 ^b^	0.57 ± 0.04 ^a^			**

^a,b,c^ = values within the same row associated with different letters are significantly different (* = *p* < 0.05; ** = *p* < 0.01), ANOVA and Tukey-HSD posthoc test. ^1^ F/C = forage to concentrate ratio, calculated on the basis of average daily feed intake per goat, evaluated by mean of dry matter intake (DMI) prediction models for lactating goats reported by Pulina et al. [30]. ^2^ = the 20:0 peak might include CLA c9t11, accounting for 0.6% of total FA in hay-fed goat milk [39] (. ^3^ AI (Atherogenic Index) = (12:0 + 4 × 14:0 + 16:0)/(n6 + n3 + MUFA). ^4^ TI (Thrombogenic Index) = (14:0 + 16:0 + 18:0)/(0.5 × MUFA + 0.5 × n6 + 3 × n3 + n3/n6) [40]. ^5^ h/H (hypocholesterolemic/Hypercholesterolemic ratio) = (18:1n9 + 18:1n7 + 18:2n6 + 18:3n6 + 18:3n3 + 20:3n6 + 20:4n6 + 20:5n3 + 22:4n6 + 22:5n3 + 22:6n3)/(14:0 + 16:0) [41].

**Table 2:** Result of the application of the AWIN (Animal Welfare Indicators) protocol, expressed as proportion of animals with no welfare problems for each specific indicator.

Farm	Proper Disbudding	Absence of Abscesses	Absence of Kneeling	Free Access at Feeding	Free Access at Drinking	Good Hair Coat Condition	Absence of Oblivion	Absence of Thermal Stress	Normal Gait
O	97.22%	100.00%	88.89%	91.67%	97.22%	97.22%	100.00%	100.00%	100.00%
M	87.50%	95.83%	12.50%	95.83%	100.00%	33.33%	100.00%	100.00%	100.00%
C	82.05%	97.44%	100.00%	92.31%	97.44%	87.18%	100.00%	100.00%	97.44%
